# Diverse Stress-Inducing Treatments cause Distinct Aberrant Body Morphologies in the *Chlamydia*-Related Bacterium, *Waddlia chondrophila*

**DOI:** 10.3390/microorganisms8010089

**Published:** 2020-01-09

**Authors:** Aurélie Scherler, Nicolas Jacquier, Carole Kebbi-Beghdadi, Gilbert Greub

**Affiliations:** Center for Research on Intracellular Bacteria, Institute of Microbiology, University Hospital Center and University of Lausanne, 1011 Lausanne, Switzerland; aurelie.scherler@chuv.ch (A.S.); nicolas.jacquier@chuv.ch (N.J.); carole.kebbi-beghdadi@chuv.ch (C.K.-B.)

**Keywords:** persistence, intracellular bacteria, division, aberrant bodies, *Chlamydiales*, *Chlamydia*-related bacteria, *Waddlia chondrophila*

## Abstract

Chlamydiae, such as *Chlamydia trachomatis* and *Chlamydia pneumoniae*, can cause chronic infections. It is believed that persistent forms called aberrant bodies (ABs) might be involved in this process. AB formation seems to be a common trait of all members of the *Chlamydiales* order and is caused by distinct stress stimuli, such as β-lactam antibiotics or nutrient starvation. While the diverse stimuli inducing ABs are well described, no comprehensive morphological characterization has been performed in *Chlamydiales* up to now. We thus infected mammalian cells with the *Chlamydia*-related bacterium *Waddlia chondrophila* and induced AB formation using different stimuli. Their morphology, differences in DNA content and in gene expression were assessed by immunofluorescence, quantitative PCR, and reverse transcription PCR, respectively. All stimuli induced AB formation. Interestingly, we show here for the first time that the DNA gyrase inhibitor novobiocin also caused appearance of ABs. Two distinct patterns of ABs could be defined, according to their morphology and number: (i) small and multiple ABs versus (ii) large and rare ABs. DNA replication of *W. chondrophila* was generally not affected by the different treatments. Finally, no correlation could be observed between specific types of ABs and expression patterns of *mreB* and *rodZ* genes.

## 1. Introduction

The *Chlamydiales* order is composed of obligate intracellular Gram-negative bacteria, which are classified either in the *Chlamydiaceae* family or in several other family-level lineages commonly referred to as “*Chlamydia*-related bacteria”. The *Chlamydiaceae* family includes three well-known human pathogens, *Chlamydia trachomatis*, causing genital tract infections or trachoma, as well as *Chlamydia pneumoniae* and *Chlamydia psittaci*, responsible for acute respiratory diseases and psittacosis, respectively [1]. 

Belonging to the *Waddliaceae* family, *Waddlia chondrophila*, a *Chlamydia*-related bacterium, is considered as a potential agent of abortion in ruminants [2,3,4] and of adverse pregnancy outcomes in humans [5,6,7,8]. Furthermore, *W. chondrophila* might also be implicated in tubal infertility [9]. Finally, *W. chondrophila* DNA was detected in nasopharyngeal aspirates from children with bronchiolitis [10] and in respiratory samples from patients with pneumonia [11], suggesting a potential role of this bacterium in respiratory diseases. 

All members of the *Chlamydiales* order share a biphasic developmental cycle beginning with adhesion and internalization of infectious nondividing elementary bodies (EBs) through phagocytosis or endocytosis [12,13]. Once inside the cells, EBs reside in a vacuole referred to as an inclusion and are converted into noninfectious dividing reticulate bodies (RBs) [14], which replicate by binary fission. Finally, RBs redifferentiate into EBs, which are released by exocytosis or by cell lysis, allowing the initiation of a new life cycle [13,15]. 

Under certain circumstances, both in vitro and in vivo, the chlamydial developmental cycle exhibits alternative forms called aberrant bodies (ABs). This form is defined as persistent, because it is a reversible, viable, nonproliferating form [16]. ABs formation can be induced by diverse stress stimuli such as addition of β-lactam antibiotics [17] or phosphomycin [18], iron or nutrient starvation [19,20], treatment with interferon-γ [21], as well as co-infection of the host cells with herpes simplex virus [22]. In presence of these diverse stress stimuli, RBs proliferation is inhibited, resulting in the formation of abnormal and enlarged bacteria. This distinct enlargement is probably due to division inhibition and continuous growth. In certain conditions, such as treatment with penicillin, it was observed that DNA replication still happened in ABs, making them multiploid. Indeed, treatment of *C. trachomatis* with penicillin induced accumulation of a minimum of 16 chromosomal copies in each AB [23]. Interestingly, when the stress stimulus is removed, ABs re-differentiate, via a poorly described mechanism, into RBs and subsequently, into infectious EBs, allowing the completion of the life cycle. ABs are thus considered as a persistent form of the bacteria, and this feature might be conserved among *Chlamydiales* as ABs have also been reported in *W. chondrophila*, *Estrella lausannensis*, and *Simkania negevensis*. Indeed, spontaneous ABs were observed in human endometrial Ishikawa cells infected with *W. chondrophila* as early as 72 h post-infection (hpi) [24]. More recently, several antibiotics and a division inhibitor have been shown to induce ABs in *W. chondrophila*-infected Vero cells [18]. Furthermore, treatment with penicillin G also induced ABs in Vero cells infected with *E. lausannensis* [25]. Finally, a small proportion of bacteria harbored enlarged morphology in *S. negevensis* treated with phosphomycin [26]. In vivo, the presence of ABs has also been reported in the intestinal enterocytes of pigs infected with *Chlamydia suis* [27] as well as in endocervix from nontreated *C. trachomatis*-infected patients [28]. ABs might thus play an important role in persistent and chronic infections by *Chlamydiales*. 

Initially, the chlamydial cell wall was hypothesized to be devoid of glycan components [29]. Surprisingly, even though no classical peptidoglycan (PG) component was detected, penicillin treatment inhibited the *Chlamydia* growth and induced the formation of ABs [30]. In addition, all enzymes required for synthesis of PG precursors as well as penicillin-binding protein homologues (Pbp), involved in the final stages of PG synthesis, are encoded in *Chlamydia* [31]. This apparent contradiction was referred to as the “chlamydial anomaly” [32]. Presence of a PG sacculus was first detected in *Protochlamydia amoebophila*, a *Chlamydia*-related bacterium, by electron cryotomography, mass spectrometry, and fluorescent labelling dyes [33]. Interestingly, in the same study, no evidence of PG was found in the *Chlamydia*-related *S. negevensis* [33]. Later on, PG was described and characterized in *C. trachomatis* using D-amino acid dipeptide probes, which successfully labelled a PG ring limited to the division plane during replication [34,35]. This indicates that different members of the *Chlamydiales* order may harbor different structures of PG, from transient PG ring at the division septum to complete sacculus. In contrast to *Chlamydiaceae*, *W. chondrophila* is resistant to β-lactam antibiotics, which bind to Pbp homologues and therefore inhibit the final steps of PG synthesis [36]. However, phosphomycin, a drug targeting MurA, the first enzyme in the PG biosynthesis pathway, blocks the growth of *W. chondrophila*, while *Chlamydiaceae* are resistant to it thanks to a point mutation in MurA [18]. 

Within most bacteria, septal PG remodeling is coordinated by the tubulin homologue FtsZ, which forms a contractile ring at the future division site and recruits cell division proteins [37]. Interestingly, no FtsZ homologue has been found among members of the *Chlamydiales* [38,39]. In absence of FtsZ, chlamydial cells apparently divide by binary fission [15,40]. FtsZ seems to be functionally replaced by MreB, an actin homologue assembling into filaments, which borders the cytoplasmic membrane in rod-shaped bacteria [41,42]. Two inhibitors of MreB, MP265 and A22, were shown to block the proliferation of *C. trachomatis*, *C. pneumoniae*, and *W. chondrophila*, indicating that MreB is required for proper chlamydial division [18,43]. Besides this growth alteration, both MreB inhibitors induced the formation of ABs [18,43]. While MreB localizes at the division septum at late stages of division, its membrane anchor RodZ is an early septal component which accumulates earlier at the division septum in *W. chondrophila* [18]. 

Despite our broad knowledge on the diverse stimuli inducing ABs in vitro, no thorough characterization of these forms has been performed up to now. In addition, precise definitions of normal RBs and ABs are lacking. To clarify these issues, *W*. *chondrophila* was used as a model organism in this study. Vero cells were infected with *W. chondrophila* and treated with diverse stimuli in order to induce the formation of ABs. Their number and morphology as well as their length and area were quantified by immunofluorescence microscopy. In addition, the impact of diverse stress stimuli on DNA replication and on the expression of two genes (*mreB* and *rodZ*), encoding for division-associated proteins, were assessed using quantitative polymerase chain reaction (qPCR) and reverse-transcription quantitative polymerase chain reaction (qRT-PCR), respectively. 

## 2. Materials and Methods 

### 2.1. Antibodies, Reagents, and Drugs

Polyclonal rabbit antibodies against *W. chondrophila* were produced in our laboratory as described previously [44]. Secondary antibody, Alexa Fluor^®^ 488 donkey anti-rabbit IgG, was purchased from Thermo Fisher Scientific (Waltham, MA, USA). Clavulanic acid, deferoxamine, mecillinam, novobiocin, penicillin, phosphomycin, piperacillin, teicoplanin, and 2,2′-bipyridyl were purchased from Sigma-Aldrich (St. Louis, MO, USA). Vancomycin was obtained from AppliChem (Darmstadt, Germany). MP265 was purchased from American Custom Chemicals Corporation (San Diego, CA, USA). All drugs were diluted in deionized water with the exception of MP265 and 2,2′-bipyridyl, which were diluted in dimethyl sulfoxide (DMSO, AppliChem) and in ethanol, respectively, in order to obtain the specific concentrations described in Table 1. Finally, the solutions were filtered through a 0.22 μm pore filter and stored at −20 °C.

### 2.2. Culture of Amoebae and W. chondrophila

*Acanthamoeba castellanii* strain (ATCC 30010) was cultured in 25 cm^2^ cell culture flasks with 10 mL of Peptone−Yeast extract−Glucose (PYG) medium (20 g/L peptone, 2 g/L yeast extract, 0.98 g/l magnesium sulphate, 1 g/L trisodium citrate, 0.02 g/L ammonium iron II sulphate, 350 mg/L monopotassium phosphate, 394 mg/L disodium phosphate, 18 g/L D-(+)-glucose, 59 mg/L calcium chloride) at 25 °C. *Waddlia chondrophila* strain ATCC VR-1470^T^ was co-cultivated with *A. castellanii* in 25 cm^2^ flasks containing 10 mL of PYG at 32 °C. After one week of incubation, cell suspension was collected and filtered through a 5 μm pore filter to retain amoebal cysts and large cellular debris. The bacterial suspension recovered from the flow-through was diluted 100 times in PYG to infect *A. castellanii* or 1000 times in Dulbecco’s modified Eagle medium (DMEM, PAN-Biotech, Aidenbach, Germany) to infect Vero cells.

### 2.3. Cell Culture and Infection Procedure

Vero cells (ATCC CCL-81) derived from kidney epithelial cells of the African Green Monkey were grown in DMEM with 10% Fetal Bovine Serum (FBS, Connectorate, Dietikon, Switzerland) in 75 cm^2^ flasks or in 24-well plates at 37 °C in presence of 5% CO_2_. Confluent cell cultures in 24-well plates were infected with *W. chondrophila* at a final dilution of 1:1000, which corresponds to a multiplicity of infection (MOI) of 5 as estimated by *W. chondrophila-*specific quantitative PCR (described in 2.8). Cells were centrifuged for 10 min at 1790× *g* at room temperature (RT), incubated for 15 min at 37 °C in presence of 5% CO_2_, and washed once with phosphate buffer saline (PBS) in order to remove nonadherent bacteria, before addition of fresh culture medium. Finally, infected cells were incubated at 37 °C in presence of 5% CO_2_. The time 0 hpi corresponds to the time when *Waddlia* suspension was added to the Vero cells monolayer. Drugs diluted at concentrations indicated in Table 1 were added at 2 hpi. Samples were harvested at different time points between 4 and 72 hpi. Cell suspension produced by scraping was used for DNA and RNA extractions, while adherent Vero cells on glass slides were used for subsequent immunofluorescence experiments.

### 2.4. Cell Viability Monitoring Using Resazurin

Vero cell viability in the presence of the diverse treatments was determined using the in vitro resazurin-based toxicology assay kit (Sigma-Aldrich). Vero cells were seeded at 2.5 × 10^4^ cells per well in 96-well plates and after an overnight incubation, were grown in presence of the drugs or 0.1% Triton X-100 for 24 h at 37 °C and 5% CO_2_. Ten percent resazurin dye solution was added to the culture, and Vero cells were further incubated during 2 to 4 h at 37 °C, 5% CO_2_. Absorbance was monitored using a FLUOstar Omega microplate reader (BMG Labtech, Offenburg, Germany) at 540 nm.

### 2.5. Propidium Iodide-Based Assay

Vero cells were seeded at a density of 4 × 10^4^ cells/well in 96-well plates. After overnight incubation at 37 °C with 5% CO_2_, cells were infected as described in Section 2.3. Propidium iodide (Sigma-Aldrich) at a concentration of 6.6 μg/mL and serial dilutions of drugs were added at 2 hpi. Vero cells treated with 0.1% Triton X-100 were considered as a positive control and untreated cells as negative control of cellular mortality. The positive control was considered as 100% of cellular mortality. Absorbance at 540 nm was measured at 24 hpi, 48 hpi, and 72 hpi with a FLUOstar Omega microplate reader (BMG Labtech). 

### 2.6. Immunofluorescence

Infected Vero cells grown on coverslips in 24-well plates were fixed with ice-cold methanol for 5 min at RT and washed three times with PBS. Cells were then blocked and permeabilized in blocking solution (PBS, 1% BSA, 0.1% saponin, 0.04% NaN3) for at least 2 h at 4 °C. Coverslips were then incubated 1 h at RT in a humidified chamber with rabbit anti-*W. chondrophila* antibodies diluted 1:1000 in blocking solution. After three washes with PBS, coverslips were incubated with a 1:1000 dilution of secondary antibodies in blocking solution containing 150 ng/mL DAPI (Invitrogen, Carlsbad, CA, USA). Alexa Fluor 488 goat anti-rabbit IgG (Thermo Fisher Scientific) was used as secondary antibody. In addition, 100 ng/mL of Texas Red-conjugated Concanavalin A (Invitrogen) was added to label host cells. Coverslips were washed three times with PBS, once with deionized water, and mounted onto glass slides using Mowiol (Sigma-Aldrich). Pictures and Z-stack images were taken by confocal microscopy using a Zeiss LSM 710 Meta (Zeiss, Oberkochen, Germany) microscope at the cellular imaging facility of the University of Lausanne (CIF). Images were then analyzed with the ImageJ software, version 1.50b (https://imagej.nih.gov). The length and area for each bacterium were measured on the Z-stack picture at which the bacterial size was maximal. In addition, overall estimation of the number of ABs per infected cell was performed using Z-stack images.

### 2.7. Quantification of Recoverable Infectious Progeny

Vero cells grown on glass coverslips were infected as described in 2.3 and treated with the corresponding drug concentrations at 2 hpi. Infected cultures were scraped at 48 hpi, and cell suspension was centrifuged at maximal speed for 10 min. The supernatant was removed, and the pellet resuspended in 1 mL SPG buffer. Samples were centrifuged at 200× *g* for 10 min, and the supernatants were stored at −80 °C. For quantification, thawed samples were serially diluted in DMEM to infect Vero cells plated on glass coverslips. Infected Vero cells were fixed and stained at 24 hpi as described in Section 2.6. Inclusions were counted in 10 random fields in duplicate by fluorescence microscopy. The number of inclusion-forming units (IFU) was then calculated and expressed as IFU per milliliter.

### 2.8. Quantitative PCR

Quantitative PCR targeting the *Waddlia* 16s rRNA gene was performed to assess bacterial growth [10]. Infected cells were harvested by scraping at 4, 8, 24, 32, 48, and 72 hpi. DNA was extracted from 50 μL of cell suspension using the Wizard SV Genomic DNA Purification System kit (Promega, Madison, WI, USA). DNA purification was conducted according to the manufacturer’s instructions with the following modifications: samples were incubated in Digestion Solution Master Mix for at least 2 h at 55 °C, and DNA elution was performed with 200 μL of water. In order to detect *W. chondrophila* 16S rRNA gene, 5 μL of DNA sample was mixed with 200 nM of forward primer WadF4, 200 nM of reverse primer WadR4, 100 nM of probe WadS2 and iTaq^TM^ Universal Probes Supermix with ROX (BioRad, Hercules, CA, USA), as described by Goy and colleagues [10] (Table S1). Cycling conditions were 3 min at 95 °C, followed by 40 cycles of 15 s at 95 °C and 1 min at 60 °C. The amplification was performed with the stepOne Plus Real-time PCR System (Applied Biosystems, Carlsbad, CA, USA). Prism 7.03 for Windows (GraphPad software, San Diego, CA, USA) was used for statistical analyzes.

### 2.9. RNA Extraction, cDNA Synthesis, and qPCR

Quantitative RT-PCR was performed to quantify gene expression of *mreB* and *rodZ*, and the 16S rRNA gene was used as an endogenous control. Primers targeting *mreB*, *rodZ*, and 16S rRNA encoding genes were previously designed in-house (Table S1) [10,18]. At 4, 8, 24, 32, 48, and 72 hpi, infected Vero cells were harvested by scraping. From the cell suspensions obtained, 500 μL was mixed with 1 mL of RNA Protect (Qiagen, Venlo, Netherlands), vortexed, and incubated for 5 min at RT. The suspensions were centrifuged for 10 min at 5000× *g*, supernatant was removed, and the pellet was then stored at −20 °C for 2 weeks or at −80 °C up to one month. RNA was extracted using the RNeasy Plus kit (Qiagen) according to the manufacturer’s protocol. DNA contamination was eliminated using the Ambion DNA-free kit^TM^ (Life technologies, Grand Island, NY, USA) according to the manufacturer’s instructions. The synthesis of first-strand cDNA was performed using the GoScript^TM^ Reverse Transcription System (Promega), whereby 4 μL of the extracted RNA and 1 μL of random primers were incubated for 5 min at 70 °C in a heating block. This sample was then added to 7.3 μL of Nuclease-Free Water, 4.0 μL of GoScript^TM^ 5X Reaction Buffer, 1.2 μL of MgCl_2_, 1 μL of PCR Nucleotide Mix, 1 μL of GoScript^TM^ Reverse Transcriptase, and 0.5 μL of Recombinant RNasin^®^ Ribonuclease Inhibitor. cDNA synthesis conditions were 5 min annealing at 25 °C, 1 h at 42 °C for extension, and finally, 15 min at 70 °C for reverse transcriptase inactivation. 

For the qPCR, 4 μL of cDNA diluted 10 times was mixed with 10 μL of iTaq Universal SYBR Green Supermix (Biorad), 4.8 μL of water, and 0.6 μL (300 nM) of each specific forward and reverse primer targeting the 16S rRNA gene (WadF4 and WadR4), *mreB*, or *rodZ*. Cycling conditions were 3 min at 95 °C, followed by 45 cycles of 15 sec at 95 °C and 1 min at 60 °C. A melting curve was then performed (15 sec at 95 °C, 1 min at 55 °C, with a +0.5 °C increment up to 65 °C, 15 sec at 95 °C). The amplification and detection of PCR products were performed with the stepOne Plus Real-time PCR System (Applied Biosystems). The fold change was calculated with the ΔΔCt method [52] and compared with untreated samples by using the 16S rRNA as an endogenous control. In more details, the following formula was used: ∆∆Ct = (Ct_gene_ − Ct_16S_)_treated_ − (Ct_gene_ − Ct_16S_)_untreated_. The fold change was then equal to 2^−∆∆Ct^. Finally, Prism 8.0.1 for Windows (GraphPad software) was used for statistical analyzes. 

## 3. Results

### 3.1. Stress Stimuli Induce Different Morphological Subtypes of Aberrant Bodies

In order to select the optimal concentrations inducing ABs, dose–response curves were performed for each drug used in this study. Vero cells were selected as host cells, since they are better suited for drug susceptibility tests than amoebae, which harbor efflux machineries that protect them from chemical compounds [36]. Moreover, several studies already used Vero cells for treatment of *W. chondrophila* with different drugs [18,53,54]. Therefore, Vero cells were infected with *Waddlia* and treated with drug concentrations ranging from 8 to 1000 μg/mL. A propidium iodide-based assay was then performed, and the percentage of mortality of Vero cells was calculated (Figure S1). Completion of the developmental cycle of *W. chondrophila* in Vero cells caused lysis and thus death of the host cells (Figure S1A). Treatment with increasing concentrations of a drug known to induce ABs formation, such as phosphomycin, caused a decrease in host cell mortality (Figure S1B). This indicates that host cell survival measurement can be used as a proxy to estimate the effect of the drugs on the proliferation of *W. chondrophila*. Dose-response curves were performed for all drugs used in this study (Figure S1C-L). According to these dose–response curves, the lowest concentrations of the drugs inducing low percentage of mortality were selected for further investigations (Figure S1, arrows). Interestingly, in some cases, high concentrations of the drugs caused increased mortality of Vero cells, indicating toxicity of the drugs. This is the case for 2,2′-bipyridyl and novobiocin and, to a less extent, for vancomycin and teicoplanin. It has to be noted that results with MP265 should be interpreted carefully due to the high toxicity of this compound.

Since *Chlamydiae* are obligate intracellular bacteria, drug treatments could indirectly affect bacteria through modification of the host cell fitness. To exclude such an indirect effect, metabolic fitness of Vero cells was measured using a resazurin test in the presence of different stress stimuli at the described concentrations. Results indicated no strong alteration in the metabolism of the eukaryotic host cells (Figure S2). Furthermore, the percentage of infected cells assessed by immunofluorescence was not strongly impacted by the presence of the stress stimuli (Table 1).

**Figure 1:**
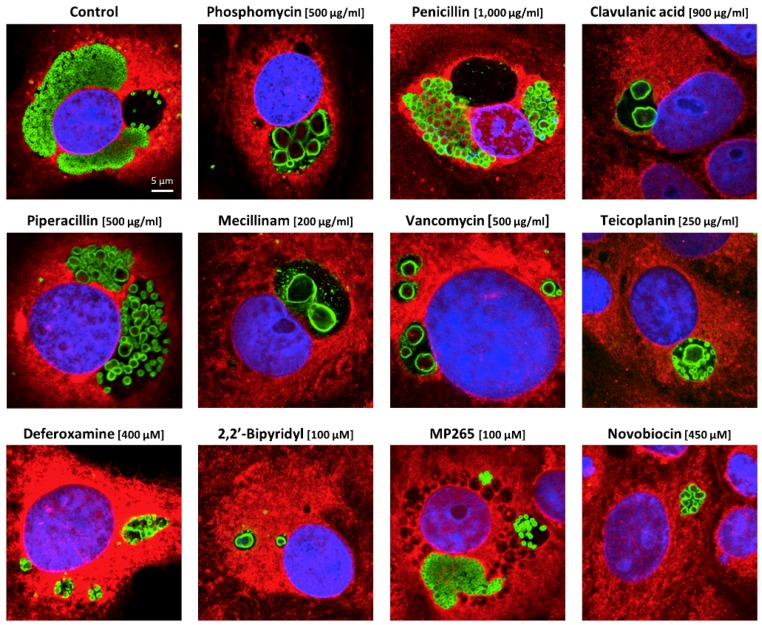
Stress stimuli induce aberrant bodies in *Waddlia-*infected Vero cells. Vero cells were infected with *W. chondrophila* and treated at 2 hpi with drugs at concentrations indicated in brackets. Vero cells were fixed with methanol at 24 hpi, labelled with concanavalin A (red), DAPI (blue), and anti-*W. chondrophila* antibodies (green) and observed by confocal microscopy. All pictures display the same scale.

In order to induce ABs, *Waddlia*-infected Vero cells were treated at 2 hpi with diverse stress stimuli at the concentrations indicated in Table 1 and labelled by immunofluorescence staining at 24 hpi. Each treatment induced the formation of enlarged bacteria (Figure 1). In order to confirm that the drug concentrations chosen were optimal, we also performed immunofluorescence staining with lower concentrations of the drugs (Figure S3). These lower concentrations failed to cause formation of inclusions filled only with ABs, confirming that concentrations chosen in Table 1 were optimal for ABs induction. In the presence of penicillin, piperacillin, or MP265 (MreB inhibitor), numerous moderately enlarged bacteria were present in each host cell (Figure 1). In contrast, phosphomycin, clavulanic acid, mecillinam, vancomycin, and teicoplanin treatments induced the formation of few but highly enlarged bacteria. When Vero cells were treated with deferoxamine, 2,2′-bipyriyl, and novobiocin, inclusions contained few moderately enlarged bacteria. With some drugs, such as teicoplanin and MP265, a mix of enlarged and normal RBs could be observed. In these cases, and in absence of a clear size cut-off to differentiate normal RBs from ABs, discrimination between the two bacterial forms was difficult. 

### 3.2. ABs Length and Area Vary According to the Stress Stimuli Applied to Infected Cells

To define the thresholds of length and area above which enlarged bacteria would be considered as ABs, 100 bacteria from untreated samples fixed 24 hpi were measured with ImageJ on confocal microscopy pictures. Thresholds were defined as the median length or area plus two standard deviations (SD). These measurements resulted in a length threshold of 1.08 μm (0.9 + 0.18) and in an area threshold of 0.79 μm^2^ (0.57 + 0.22) (Table 1). Above these limits, bacteria were considered as ABs. As previously published, the growth kinetic of *W. chondrophila* in Vero cells indicates that bacteria are in the replicative phase at 24 hpi and that inclusions are filled with RBs at that time postinfection [24]. However, since the presence of some EBs could not be entirely excluded, bacteria below the calculated thresholds were defined as small bodies (SBs). The limits of detection for SBs were set at 0.6 μm for length and 0.4 μm^2^ for area. For all treated samples, median length and area of 100 bacteria were calculated (Table 1). Length and area medians were higher in presence of phosphomycin, penicillin, clavulanic acid, piperacillin, mecillinam, and 2,2′-bipyridyl, consistent with the presence of large ABs inside these inclusions. With all other drugs, the length and area medians were closer to the thresholds between ABs and SBs. Using the same data and the previously defined cut-offs, we calculated the percentage of ABs among these 100 bacteria as well as their median length and area (Table 1). In presence of phosphomycin, penicillin, clavulanic acid, piperacillin, mecillinam, deferoxamine, and 2,2′-bipyridyl, more than 90% of the bacteria were classified as ABs (100% for phosphomycin). In contrast, upon treatments with vancomycin, teicoplanin, MP265, and novobiocin, percentages of ABs were lower, confirming our previous observation that inclusions are essentially filled with a mix of ABs and SBs (Figure 1). As expected, the median lengths and areas were slightly higher when SBs were removed from the data set. 

**Figure 2:**
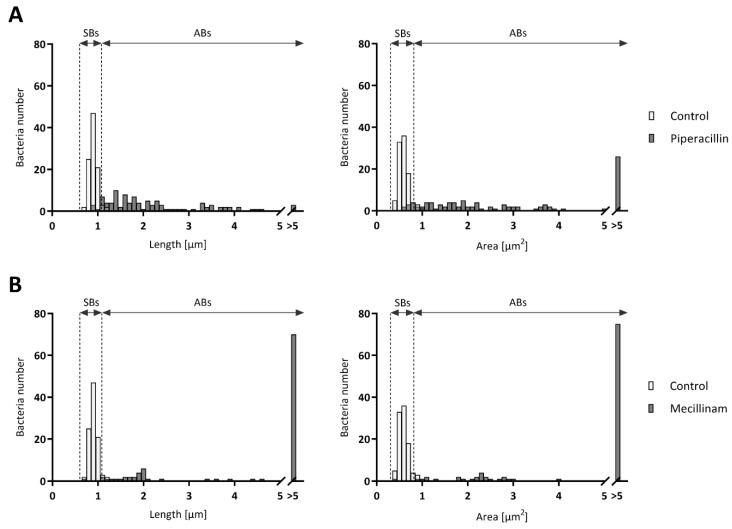
Frequency distribution of bacterial length and area. *W. chondrophila*-infected Vero cells were treated at 2 hpi with 500 µg/mL piperacillin (**A**) or mecillinam (**B**) and harvested at 24 hpi. Cells were then treated for immunofluorescence and imaged by confocal microscopy. The maximum length and area of 100 bacterial particles were measured with ImageJ. The limits of detection for SBs (small bodies), indicated with the first dotted line, were set at 0.6 μm for length and 0.4 μm^2^ for area. The cut-off between normal SBs and ABs is indicated by the second dotted line. The difference of frequency distribution between untreated and treated conditions, in terms of maximum length and area, was significant for both drugs with a *p* value of <0.0001 using the Mann–Whitney test.

Frequency distribution analyses were then performed on bacteria length and area values measured in each treated condition (Figure 2 and Figure S4). Interestingly, significant distinctions can be noticed between control and treatments with piperacillin or mecillinam (Figure 2A,B). Piperacillin treatment induced smaller ABs as previously observed on confocal microscopy pictures. In the presence of mecillinam, the length of almost 70% of the bacteria was above 5 μm and nearly 80% of the bacteria displayed an area above 5 μm^2^, indicating the presence of very large ABs. With phosphomycin (Figure S4A), penicillin (Figure S4B), clavulanic acid (Figure S4C), vancomycin (Figure S4D), deferoxamine (Figure S4F), and 2,2′-bipyridyl (Figure S4G), significant differences of frequency distribution were observed between control and treated conditions. In contrast, these differences were less obvious, but remained significant with teicoplanin (Figure S4E), MP265 (Figure S4H), and novobiocin (Figure S4I) treatments, which again confirms the presence of a mix of normal SBs and ABs inside these inclusions. To summarize, all stress-inducing conditions tested caused the formation of ABs, that is, bacteria defined by a higher length and larger area than the ones observed in control conditions. However, the morphology of these ABs varies according to the type of stress stimuli applied to the infected cells. 

### 3.3. Number of ABs Per Infected Cell and Inclusion Composition Vary with the Different Stress Stimuli

**Figure 3:**
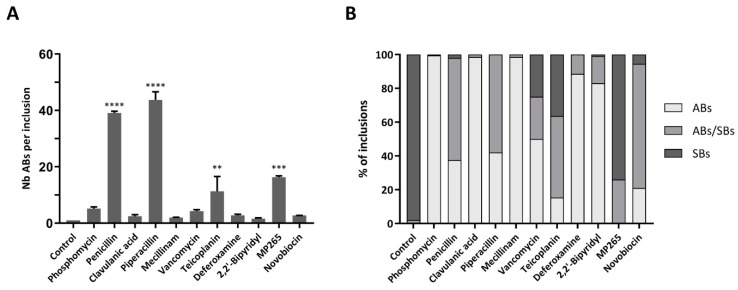
Effects of the stress stimuli on the number of ABs per infected cells and on the inclusion composition at 24 hpi. *W. chondrophila*-infected Vero cells were treated at 2 hpi with the indicated drugs at concentrations indicated in Table 1 and then processed as in Figure 2. (**A**) Mean number of ABs per inclusion according to the stress stimuli treatment. The results show the mean ± SD of 50 infected cells from one representative experiment counted in duplicate. Number of ABs were counted on z-stack images of confocal microscopy. Significant differences between control and the other treatments determined with the one-way analysis of variance (ANOVA) are indicated by an asterisk (* *p* < 0.05, ** *p* < 0.01, *** *p* < 0.001, **** *p* < 0.0001). (**B**) Percentage of inclusions containing ABs only, ABs and SBs, or SBs only. From one representative experiment, 200 inclusions were counted.

Using the cut-off defined above, the number of ABs per inclusion cells was counted for each condition at 24 hpi (Figure 3A). In the presence of penicillin and piperacillin, we could estimate an average of 40 ABs per inclusion. Phosphomycin, clavulanic acid, mecillinam, vancomycin, deferoxamine, 2,2′-bipyridyl, and novobiocin treatments induced between 2 and 5 ABs per inclusion. In contrast, teicoplanin and MP265, caused an intermediate phenotype with a mean of about 15 ABs per inclusion. Only very few ABs were present in untreated samples, corresponding to less than one AB per inclusion. To perform statistical comparisons between the different phenotypes, number of ABs induced by each drug was compared with control. Statistical analysis confirmed the presence of distinct phenotypes in terms of AB numbers. 

Finally, the proportion of inclusions containing SBs only, ABs only, or a mix of both bacterial types was assessed in infected cells treated with the different stress stimuli. Interestingly, this proportion varies according to the drug used (Figure 3B). In presence of phosphomycin, clavulanic acid, mecillinam, deferoxamine, and 2,2′-bipyridyl, a majority of inclusions contained only ABs. In contrast, novobiocin treatment induced the formation of inclusions containing principally a mix of ABs and SBs. When infected Vero cells were treated with penicillin and piperacillin, about half of the inclusions contained only ABs, whereas the other half displayed ABs and SBs. Vancomycin and teicoplanin treatments exhibited all three types of inclusions. Finally, most of the inclusions were filled with SBs only when infected Vero cells were treated with MP265. According to these results, the number of ABs per infected cell as well as the inclusion composition vary with the different stress stimuli applied to the infected cells. However, penicillin and piperacillin treatments have similar effects on the inclusion composition as well as on the number of ABs per infected cell. In addition, these similarities in terms of proportion and number can also be observed with phosphomycin, mecillinam, deferoxamine, and 2,2′-bipyridyl.

### 3.4. Treatment with the Stress Stimuli Reduces the Production of Infectious EBs

**Figure 4:**
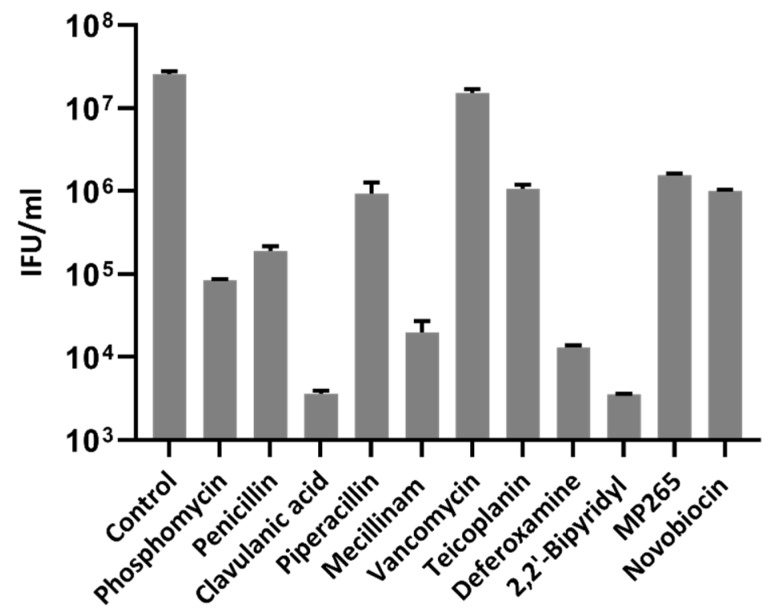
Stress stimuli decrease production of infectious elementary bodies (EBs). *Waddlia*-infected Vero cells were treated at 2 hpi with drug concentrations listed in Table 1. Infected cell lysates were collected at 48 hpi and used for infectious titers analysis. Results are presented as IFU per milliliter of inoculum. The limit of detection corresponds to one inclusion for 500 Vero cells, which represents a calculated value of approximately 10^3^ IFU/mL. Error bars represent the SD of duplicates from one representative experiment. The difference of IFU/mL was significant with a *p* value of <0.0001 determined by one-way ANOVA followed by Dunnett’s test for multiple comparisons of control with drugs. IFU: inclusion-forming units.

Persistent bacteria are considered as noninfectious [16,17]. Presence of ABs inside inclusions, as observed by immunofluorescence, should thus impact the production of infectious EBs. To confirm this hypothesis, *Waddlia-*infected cells were treated at 2 hpi with the drugs and harvested at 48 hpi. The cell suspension was then used to perform titration analyzes and to calculate IFU per milliliter. As expected, a significant reduction of recoverable IFU was observed for all treatments compared with untreated infected cells, indicating decreased infectivity (Figure 4). Treatments such as vancomycin, teicoplanin, or MP265, which induce mixed ABs/SBs or numerous ABs per inclusion, displayed higher IFU/mL than drugs inducing only few ABs, such as clavulanic acid, mecillinam, or iron chelators.

### 3.5. DNA Replication is Differentially Affected in Presence of Stress Stimuli

**Figure 5:**
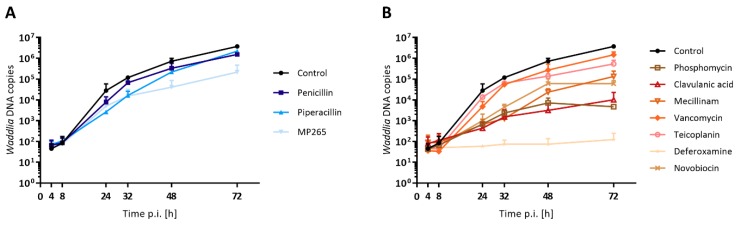
Stress stimuli treatments differentially affect *W. chondrophila* DNA replication. Vero cells were infected with *W. chondrophila*, and drugs were added at 2 hpi at concentrations indicated in Table 1. DNA was extracted at the indicated time points, and number of *Waddlia* DNA copies were quantified by qPCR. Error bars represent the SD of two independent experiments. Stress stimuli were grouped according to the ABs subtypes: (**A**) numerous and small ABs and (**B**) few and larger ABs. For each treatment, values at 48 and 72 hpi are significantly different (*p* < 0.05) to the control condition, as determined by the one-way ANOVA test.

**Figure 6:**
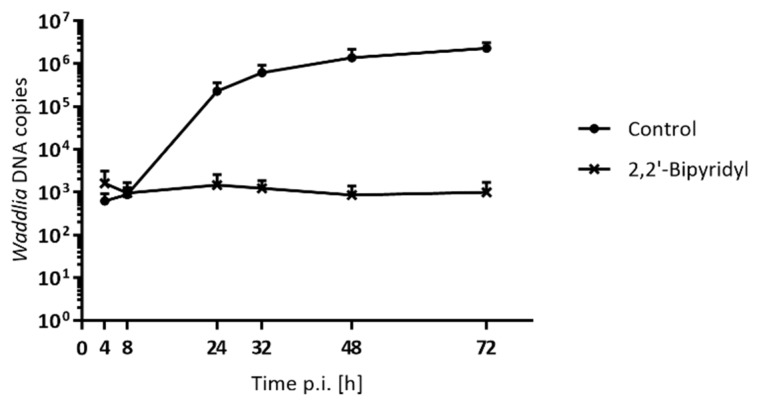
Treatment with 2,2′-bipyridyl impacts *Waddlia* DNA replication. Number of DNA copies was quantified by qPCR in *Waddlia*-infected Vero cells treated with 100 µM 2,2′-bipyridyl at 2 hpi. Error bars represent the SD of two independent experiments. Values at 48 and 72 hpi in presence of 2,2′-bipyridyl are significantly different to the untreated condition, as determined by the one-way ANOVA test.

Since *C. trachomatis* ABs were shown to contain a large number of genome copies, we wondered if the different subtypes of *W. chondrophila* ABs would differ in their DNA content. DNA replication was quantified by a qPCR targeting the 16S rRNA gene in *Waddlia*-infected Vero cells treated with different stress stimuli at 2 hpi (Figure 5). Penicillin, piperacillin, MP265, mecillinam, vancomycin, and teicoplanin treatments slightly affected the DNA replication (Figure 5A). In contrast, the number of *Waddlia* DNA copies in the presence of phosphomycin, clavulanic acid, and novobiocin reached a plateau at 48 hpi (Figure 5B), suggesting that DNA replication might be partially inhibited by these stress stimuli. Finally, both iron chelators, deferoxamine, and 2,2′-bipyridyl strongly inhibited DNA replication (Figure 5B and Figure 6).

### 3.6. Number of Genome Copies per AB with Phosphomycin and Mecillinam

Thanks to immunofluorescence experiments, we could evaluate the percentage of infected cells at 24 hpi (Table 1). With this number and the qPCR results, we could estimate the number of *Waddlia* DNA copies per infected cell. When cells were treated with phosphomycin or mecillinam, inclusions were essentially filled with ABs, and the mean number of ABs per infected cell was calculated (Figure 3A). Subsequently, the number of genome copies per bacterium could be estimated by dividing the number of DNA copies per infected cell by the number of ABs in each infected cell. We estimated the numbers of genomes to be four copies per AB for phosphomycin and six for mecillinam. Since all other treatments produced inclusions filled with a mix of ABs and SBs (Figure 3B), the number of genome copies per AB could not be calculated in these situations. 

### 3.7. Stress Stimuli Differently Modulate the Expression of mreB and rodZ

Since ABs formation apparently involves division inhibition, we investigated the expression of two genes coding for proteins required for bacterial division, MreB, an actin homologue, and its regulator RodZ. In order to determine the modulation of *mreB* and *rodZ* transcription upon ABs formation, Vero cells were infected with *W. chondrophila*, and the diverse stress stimuli were added at 2 hpi. The expression of *mreB* and *rodZ* was then measured at selected time points by qRT-PCR using specific primers (Table S1). The fold change between treated and untreated samples was calculated, and significant up or downregulations are highlighted in bold green or red, respectively (Table 2). In the presence of phosphomycin, penicillin, vancomycin, deferoxamine, and 2,2-bipyridyl, the expression of *mreB* was downregulated at nearly all time points. With piperacillin and MP265, *mreB* expression was significantly downregulated only at 48 hpi, while with novobiocin, this downregulation occurred at 12 hpi. An increase of *mreB* expression could only be observed with teicoplanin treatment at 36 hpi. Interestingly, while *mreB* expression was downregulated with vancomycin, *rodZ* expression was upregulated upon treatment with this drug. Downregulation of *rodZ* was observed at 2 or 3 time points with piperacillin, deferoxamine, MP265, novobiocin, and 2,2′-bipyridyl, and only at 48 hpi with phosphomycin. Taken together, these results indicated that there were no significant transcription variations of *mreB* and *rodZ* with clavulanic acid, mecillinam, and teicoplanin treatments at any time points.

## 4. Discussion

ABs have been described in *Chlamydiaceae* family members such as *C. trachomatis* or *C. pneumoniae* [17,19,55], and in several *Chlamydia*-related bacteria, including *W. chondrophila*, *E. lausannensis* or *S. negevensis* [18,25,26]. Morphologically, ABs are described as enlarged bacteria, probably due to division inhibition and continuous growth. However, these aberrant forms of the bacteria have not yet been precisely characterized, and the mechanisms behind their formation remain unclear.

To address this issue, several antibiotics and chemicals were chosen in this study for their potential effect on AB formation in *Waddlia-*infected Vero cells. These stress stimuli were selected because they affect various targets such as cell wall biosynthesis, DNA replication, or iron availability. Based on propidium iodide assay and immunofluorescence staining, we selected the minimum concentration inducing only ABs inside inclusions for each drug. However, concentrations used with teicoplanin, vancomycin, and MP265 failed to induce homogenous AB formation. Higher doses would have been toxic for *Waddlia*-infected Vero cells based on propidium iodide results. Therefore, we cannot exclude that observed phenotype might be due to an incomplete activity of the drug.

Using a resazurin test, we could exclude that antibiotics and chemicals used in this study induced any significant changes in host cell viability or metabolic fitness, thus confirming that ABs would be induced directly by the different stimuli and not by an alteration of the cell fitness. In presence of penicillin, the viability rate of Vero cells measured with the resazurin test might appear quite low. However, the error bars were high and no impact on the Vero cells morphology was observed on any immunofluorescence images. Furthermore, penicillin as well as MP265 were previously used to study the division in *W. chondrophila*, and the cell viability was not impacted [18]. Therefore, we can exclude that the concentration of penicillin we used (1000 μg/mL) would be toxic for the host cell. All treatments induced the formation of ABs in *Waddlia*-infected Vero cells at 24 hpi, confirming the results obtained in previous studies on *Chlamydia*-related bacteria and on *Chlamydiaceae*. Indeed, treatments with various β-lactam antibiotics, including penicillin and piperacillin, were shown to induce ABs in HeLa cells infected with *C. trachomatis* [17]. The presence of ABs with clavulanic acid is counterintuitive as this drug is normally used as a β-lactamase inhibitor in order to increase the efficacy of the β-lactam antibiotics [46]. However, high concentrations of clavulanic acid have also been reported to bind Pbps and more specifically, Pbp2 in vitro [56]. Treatment with clavulanic acid can induce ABs in *C. trachomatis*, probably by binding Pbp2 of the bacterium [17]. As Pbp2 is conserved in *W. chondrophila* [45], we think that clavulanic acid might also bind to Pbp2, leading to the formation of ABs. In addition, ABs have been reported in Vero cells infected with *W. chondrophila* and treated with numerous stress stimuli such as phosphomycin, mecillinam, vancomycin, teicoplanin, and MP265 [18]. In contrast to *W. chondrophila*, no ABs were induced when *Chlamydia* species were treated with phosphomycin. This difference is explained by a mutation in the phosphomycin target, the enzyme MurA, which confers resistance to phosphomycin [57]. In addition, ABs induced by deferoxamine or 2,2′-bipyridyl, both iron chelators, have been reported in *C. trachomatis* [19,50]. Finally, it is the first time to our knowledge that ABs are reported upon incubation with novobiocin, a drug targeting the DNA gyrase.

During our investigations, we realized that some drugs induced formation of small ABs that might be interpreted as slightly enlarged SBs. We thus decided to define clear cut-offs to discriminate normal SBs from ABs. We based our definition of these two bacterial forms on their length and area and calculated a threshold above which an enlarged SB is called an AB. According to the number of ABs and to their morphology, two main phenotypes can be observed in treated cells: (i) small and multiple ABs versus (ii) large and few ABs. The difference between these subtypes might be explained by the different mechanisms of action of the stress stimuli, which therefore can elicit various responses in *W. chondrophila*. For example, phosphomycin inhibits the first steps of peptidoglycan (PG) synthesis by targeting MurA. Therefore, in presence of this drug, the division might be blocked directly while DNA replication can continue, leading to the formation of few and large ABs. In contrast, both penicillin and piperacillin inhibit the last steps of PG synthesis by targeting Pbp2/Pbp3 [45] or Pbp3 [47], respectively, and both induced numerous and small ABs. Nevertheless, only few and large ABs were induced with mecillinam, a drug targeting Pbp2 [48]. Ouellette et al. also reported that inhibition of Pbp2 or Pbp3 results in distinct AB morphologies in *C. trachomatis*, indicating that function of both Pbps are required for proper bacterial cell division [43]. Interestingly, even if a putative class-C β-lactamase was identified in *W. chondrophila*, mecillinam was not inactivated [39]. Similar results were obtained in *E. coli* strains producing a class C β-lactamase. The inability to inactivate mecillinam might be due to the poor affinity of *E. coli* β-lactamase for mecillinam, coupled with a relatively high resistance to hydrolysis [58]. A similar mechanism is likely at play for *Waddlia*. In contrast to mecillinam, it is possible that penicillin and piperacillin are degraded by the putative class-C β-lactamase in *W. chondrophila*. This possible degradation might explain why numerous and small ABs are observed upon treatment with penicillin or piperacillin, while only few and large ABs are induced by mecillinam. In contrast, *C. trachomatis* does not encode β-lactamase enzymes, and only few and very large ABs are induced when this bacterium is treated with penicillin or piperacillin [43]. In order to determine if the difference in ABs morphology between *W. chondrophila* and *C. trachomatis* is indeed due to the β-lactamase of *W. chondrophila*, a detailed characterization of the specificity and mechanism of action of this β-lactamase should be performed in the future. Furthermore, vancomycin and teicoplanin, two glycopeptide antibiotics, induce generally large ABs. Few and small ABs were observed in presence of novobiocin, suggesting that DNA replication is not required for AB formation. However, no link between an AB subtype and a class of stress stimuli could be defined according to these results. This indicates that ABs might be formed by several distinct mechanisms depending on the stress stimulus applied, which subsequently causes different sizes of ABs. This could explain why no common genetic markers for this chlamydial form have been found yet, since several distinct forms of ABs may exist. Indeed, numerous studies have investigated how stress-inducing treatments affect the expression patterns of numerous chlamydial genes compared with untreated cells [59,60,61]. Nevertheless, only two genes, *euo* and *omcB*, have been proposed as potential markers of persistence so far, although discrepancies were found between different studies [62]. Therefore, further experiments are needed to reveal how different stress stimuli can induce distinct AB subtypes and the exact mechanisms behind the ABs formation.

ABs are commonly considered as noninfectious, and the reduction of infectious progeny in *C. psittaci* when treated with penicillin had already been reported by the early 1960s [63]. More recently, reduced infectivity by >95% was observed in *C. trachomatis* exposed to different β-lactam antibiotics [17]. Similar observations were described in *C. trachomatis* treated with the iron chelators deferoxamine and 2,2′-bipyridyl [50]. Consistently, we could observe a significant decrease in the production of infectious EBs with all treatments used in this study. However, this decrease was less important with drugs that caused only partial induction of ABs, such as teicoplanin, vancomycin, and MP265. The presence of higher IFU/mL might be explained by the presence of noninternalized EBs in the extracellular medium or at the surface of the Vero cells. For vancomycin, we hypothesize that the permeability of the antibiotic is not optimal, which might explain the presence of SBs inside inclusions and higher IFU/mL. Indeed, vancomycin, due to its molecular mass, is unable to diffuse through the ß-barrel porins of the Gram-negative bacteria [64,65]

Although the various stress stimuli clearly affect the bacterial morphology, DNA replication of *W. chondrophila* was not strongly altered, except following treatment with drugs targeting MurA (phosphomycin) or GyrB (novobiocin), with iron chelators (deferoxamine and 2,2′-bipyridyl), and with an inhibitor of MreB (MP265). Interestingly, in the presence of phosphomycin, *Waddlia* DNA replication reached a plateau at 48 hpi, and the number of DNA copies even decreased by later time point (72 hpi), which suggests that ABs, including their DNA, might be degraded by the host cell. Novobiocin was selected for its direct action on DNA replication, but interestingly, the number of DNA copies was not strongly impacted except at later time points. This result suggests that novobiocin might be insufficient to effectively block DNA replication and might thus be active on chlamydial proliferation by blocking DNA supercoiling, which then affects gene expression [51], or by affecting plasmid inheritance. Indeed, this compound was used as an effective plasmid curing agent in chlamydiae [66,67]. It has to thus be investigated whether ABs induced by novobiocin are directly due to the DNA gyrase activity of novobiocin or to its potential plasmid-curing effect. In the case of deferoxamine and 2,2′-bipyridyl, the number of genome copies remained low during the whole experiment. The depletion of iron by both chelators might trigger a check-point, causing an arrest in the developmental cycle of *W. chondrophila*, thus explaining the inhibition of DNA replication. Indeed, iron availability is essential for chlamydial proliferation, since the modulation of iron availability was shown to impact the normal life cycle of *C. pneumoniae* [68]. As observed in previous studies, DNA replication rate was altered in presence of MP265. Indeed, MreB inhibition by MP265 was shown to block the proliferation of *C. trachomatis*, *C. pneumoniae*, and *W. chondrophila*, which indicates the requirement of MreB for proper chlamydial division [18,43].

Previous studies have revealed accumulation of genomic DNA in *C. trachomatis* treated with different β-lactam antibiotics [17,23]. This tendency was also confirmed in *W. chondrophila* with an accumulation of four and six genome copies per bacterium in the presence of phosphomycin and mecillinam, respectively. These results are consistent with the assumption that, in certain conditions, DNA replication still continues in ABs, making them polyploid. However, only a few studies have investigated the possible polyploidy of ABs induced by different stimuli in *Chlamydiales*. Future investigations are needed to assess whether accumulation of chromosomal copies occurs upon treatment with defined stimuli and if the process of polyploidy is similar in host cells infected with different *Chlamydiales* such as *C. trachomatis*, *C. psittaci*, or *W. chondrophila*.

As mentioned earlier, ABs formation might be due to an inhibition of division with ongoing growth. Therefore, the expression of two division genes, *mreB*, an actin homologue, and its regulator *rodZ*, was investigated upon AB induction. Different patterns of expression were induced by the stress stimuli. However, we could not observe any correlation between the different patterns of *mreB* and *rodZ* expressions and a specific subtype of ABs. Further investigations need to be performed to investigate the expression of genes coding for other proteins implicated in the division process, such as the Tol-Pal complex, AmiA [45], or SpoIID [53]. Nevertheless, these results confirm the current consensus, which affirms that the transcriptional response of the *Chlamydiales* varies according to the stress stimulus applied.

## 5. Conclusions

In conclusion, according to the results presented here, the presence of distinct AB subtypes appears to reflect different mechanisms of AB formation, with or without inhibition of DNA replication. Interestingly, no correlation between drug target, DNA replication, *mreB* and *rodZ* expressions, or phenotype was found, which argues against a single common mechanism for AB formation. Therefore, different chemicals might cause the formation of different classes of ABs. Indeed, distinct phenotypes were also observed in *C. trachomatis* treated with different penicillin derivatives [43]. Distinct AB phenotypes might be explained by different transcriptional responses according to the stress stimuli applied. In addition, each drug might elicit a persistent state according to the cell line and the species used during the experiments. Therefore, the discovery of universal genetic markers for persistence remains quite challenging, but the development of new technologies in proteomic and transcriptomic fields will certainly provide new insights. Further studies are now necessary to discover the molecular mechanisms involved in AB formation. Indeed, a better knowledge of biological mechanisms triggering the development of ABs might be of importance to reduce treatment failures when dealing with chronic chlamydial infections.

## Figures and Tables

**Table 1:** Summary of the drugs used in the study and of their effects on the percentage of infected cells as well as on length and area of induced aberrant bodies at 24 hpi.

Drug	Class	Target	Concentration	Infected Cells [%]^a^	Length [µm]^b^*	Area [µm^2^ ]^b^*	ABs [%]^c^	ABs Length [µm]^d^	ABs Area [µm^2^]^d^
Control	/	/	/	13	0.90	0.57	2	1.17	0.89
Phosphomycin	Other	MurA [18]	500 µg/mL	19	4.24	10.73	100	4.24	10.73
Penicillin	Penicillin	Pbp2/Pbp3/FtsI/AmiA [45]	1000 µg/mL	10	2.11	2.64	98	2.11	2.78
Clavulanic acid	β-lactamase inhibitor	β-lactamase [46]	900 µg/mL	19	3.10	6.00	99	3.10	6.15
Piperacillin	Penicillin	Pbp3/(FtsI) [47]	500 µg/mL	13	1.84	2.24	93	1.93	2.57
Mecillinam	Broad-spectrum penicillin	Pbp2 [48]	200 µg/mL	13	6.65	26.48	99	6.69	26.54
Vancomycin	Glycopeptide	D-Ala-D-Ala moiety of NAM/NAG [49]	500 µg/mL	48	1.47	1.26	64	2.63	4.74
Teicoplanin	Glycopeptide	D-Ala-D-Ala moiety of NAM/NAG [49]	250 µg/mL	13	1.30	1.18	76	1.49	1.44
Deferoxamine	Iron chelator	Binds Fe^3+^ [50]	400 µM	17	1.78	1.80	92	1.80	1.90
2,2’-Bipyridyl	Iron chelator	Binds Fe^2+^ and Fe^3+^ [50]	100 µM	25	2.26	3.10	93	2.29	3.24
MP265	Other	Inhibitor of MreB [43]	100 µM	19	1.01	0.73	29	1.26	1.05
Novobiocin	Aminocoumarin	DNA gyrase subunit B (GyrB) [51]	450 µM	13.5	1.21	0.98	59	1.38	1.26

^a^ 100 cells were counted in duplicate at 24 hpi. ^b^ Length and area represent the median of 100 bacteria. ^C^ The percentage of bacteria (among the 100) above the thresholds of 1.08 μm for length and 0.79 μm^2^ for area. ^d^ ABs length and area represent the median of bacteria above the threshold. * Significant *p* value (<0.0001) between untreated and treated conditions for length and area with the Mann−Whitney test. Pbp: Penicillin-binding proteins; D-Ala-D-Ala: D-alanyl-D-alanine; NAM/NAG: N-actelymuramic acid/N-acetylglucosamine.; ABs: aberrant bodies; hpi: hours post-infection.

**Table 2:** Temporal fold change of *mreB* and *rodZ* genes in *W. chondrophila* in presence of diverse stress stimuli^a^.

	*mreB*				*rodZ*			
	12 hpi	24 hpi	36 hpi	48 hpi	12 hpi	24 hpi	36 hpi	48 hpi
Phosphomycin	0.47 ± 0.17	**0.34 ± 0.03**	**0.37 ± 0.06**	**0.14 ± 0.01**	0.72 ± 0.17	0.54 ± 0.01	0.88 ± 0.12	**0.23 ± 0.02**
Penicillin	**0.43 ± 0.00**	0.71 ± 0.51	0.86 ± 0.64	**0.24 ± 0.19**	0.95 ± 0.00	0.89 ± 0.43	1.04 ± 0.51	0.33 ± 0.23
Clavulanic acid	1.43 ± 0.40	1.32 ± 0.10	1.30 ± 0.30	1.12 ± 0.05	1.12 ± 0.17	1.95 ± 0.80	2.37 ± 0.41	0.53 ± 0.11
Piperacillin	0.47 ± 0.05	0.50 ± 0.03	0.59 ± 0.21	**0.15 ± 0.01**	**0.39 ± 0.06**	**0.36 ± 0.08**	0.47 ± 0.23	**0.13 ± 0.02**
Mecillinam	1.46 ± 0.10	0.76 ± 0.12	0.65 ± 0.03	1.78 ± 0.00	1.08 ± 0.26	1.06 ± 0.14	1.81 ± 0.11	0.88 ± 0.14
Vancomycin	0.43 ± 0.12	**0.40 ± 0.08**	**0.41 ± 0.07**	**0.24 ± 0.04**	**3.20 ± 0.04**	**2.99 ± 0.29**	**2.97 ± 0.95**	1.98 ± 0.37
Teicoplanin	0.95 ± 0.01	1.15 ± 0.02	0.93 ± 0.07	0.83 ± 0.07	1.11 ± 0.19	1.11 ± 0.16	0.96 ± 0.06	0.59 ± 0.07
Deferoxamine	0.42 ± 0.16	**0.21 ± 0.05**	**0.16 ± 0.05**	**0.04 ± 0.00**	0.64 ± 0.20	**0.31 ± 0.08**	0.49 ± 0.17	**0.09 ± 0.12**
2,2’-Bipyridyl	0.64 ± 0.04	**0.34 ± 0.14**	0.42 ± 0.34	**0.15 ± 0.05**	**0.20 ± 0.02**	**0.34 ± 0.14**	0.54 ± 0.18	**0.15 ± 0.05**
MP265	0.50 ± 0.17	0.62 ± 0.31	1.06 ± 0.06	**0.14 ± 0.00**	**0.35 ± 0.10**	0.52 ± 0.15	0.77 ± 0.09	**0.10 ± 0.01**
Novobiocin	**0.06 ± 0.06**	0.93 ± 0.39	1.20 ± 0.12	0.80 ± 0.00	**0.04 ± 0.03**	0.80 ± 0.12	1.56 ± 0.43	**0.44 ± 0.00**

^a^ The fold change was calculated using the ∆∆Ct method compared with untreated samples. The values represent the mean fold change ± SD of duplicates from two independent experiments. Red values, significant gene downregulation (fold change < 0.5); green values, significant gene upregulation (fold change >2).

## Supplementary Materials

Supplementary materials can be found at https://www.mdpi.com/2076-2607/8/1/89/s1.
