# Supplementary material for: Diverse Stress-Inducing Treatments cause Distinct Aberrant Body Morphologies in the Chlamydia-Related Bacterium, Waddlia chondrophila
Source: Microorganisms. 2020 Jan 9;8(1):89. doi: 10.3390/microorganisms8010089 (PMC7022761; doi:10.3390/microorganisms8010089)
Supplement: Supplementary file 1 [file microorganisms-08-00089-s001.zip › Scherler_Supplementary_Data_Revised/Scherler_Supplementary_Data_Revised.docx]

**Supplementary Figures and Table**

**
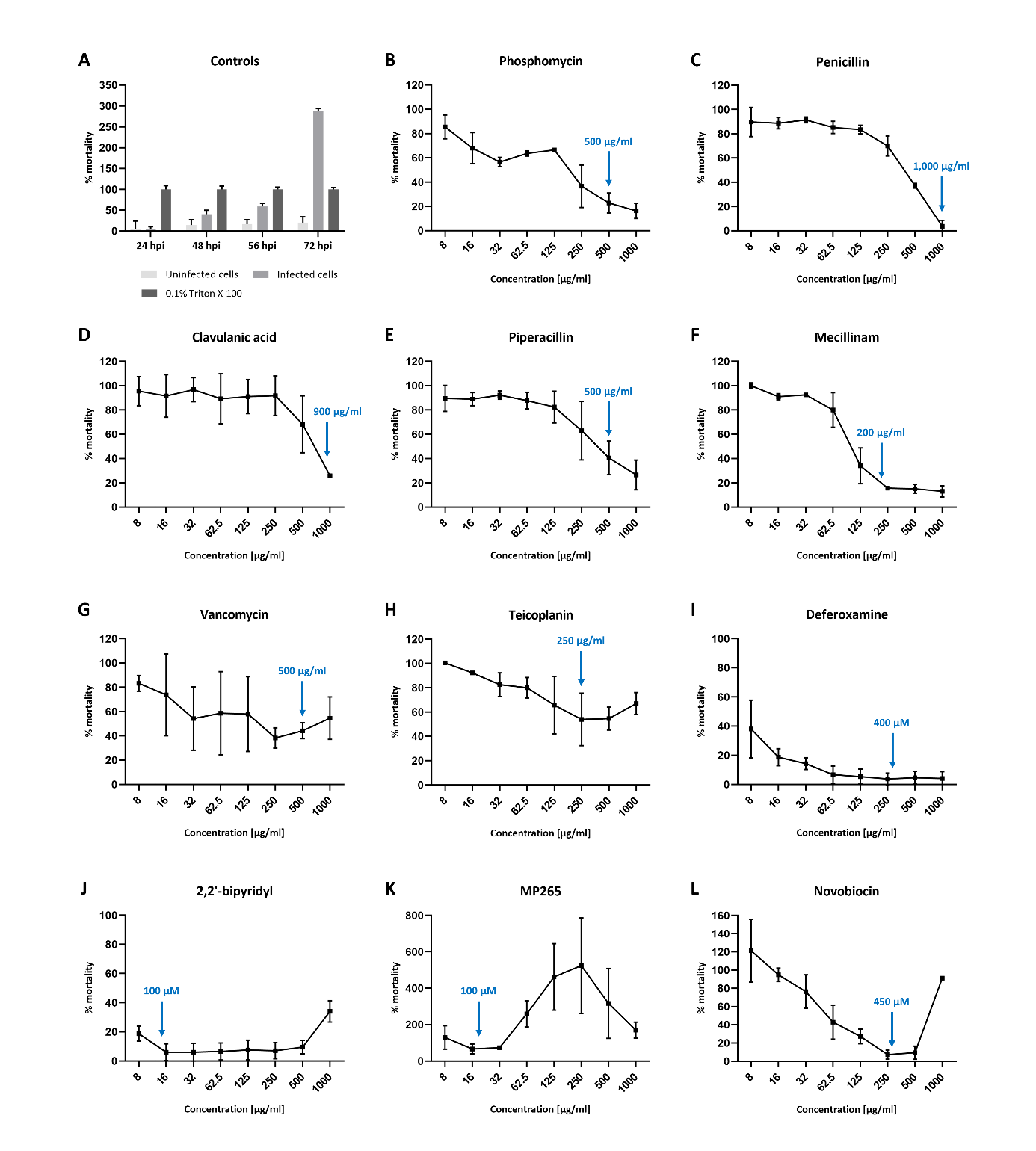
**

**Figure S1.** Percentage of mortality in *Waddlia* infected Vero cells when treated with serially diluted drugs. The cell mortality was determined by propidium iodide-based assay at 24, 48, 56 and 72 hpi for the controls (A), and at 72 hpi for the drugs (B-L). The cell mortality was calculated in percent compared to 0.1% Triton X-100 treated cells for the controls and to infected cells for the drugs. Treatment with Triton X-100 was used as a positive control of mortality. Error bars represent the SD of two independent experiments. Blue arrows indicate the concentrations used in the study.


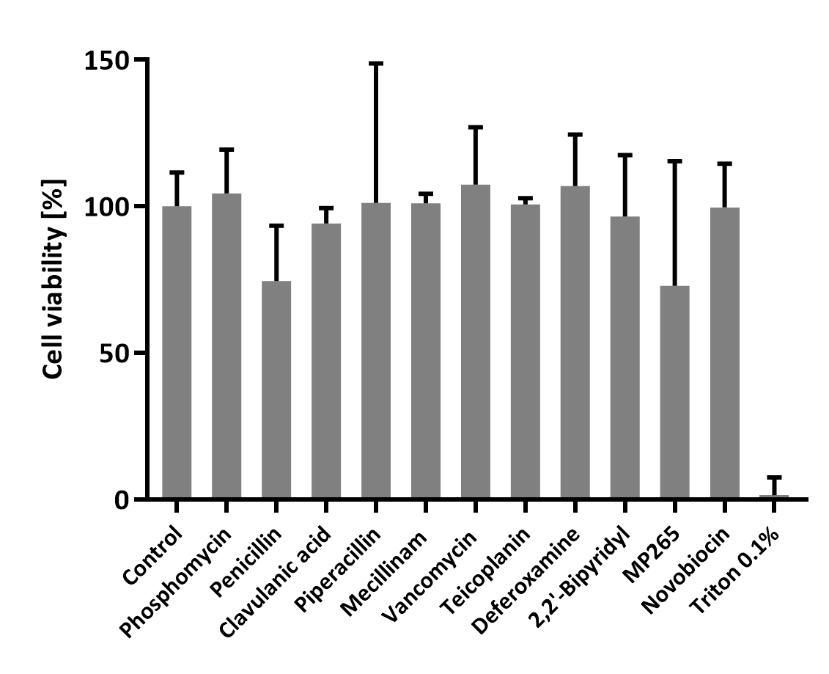


**Figure S2.** Drug treatments do not strongly affect the viability of Vero cells. Vero cells were treated for 24 h with drug concentrations indicated in Supplementary Table 1. The cell viability was then determined using Resazurin test as described and calculated in percent compared to untreated cells (control). Triton X-100 at 0.1% was used as a positive control of mortality. The graph represents the mean values ± SD of quadruplicates from one representative experiment.

**
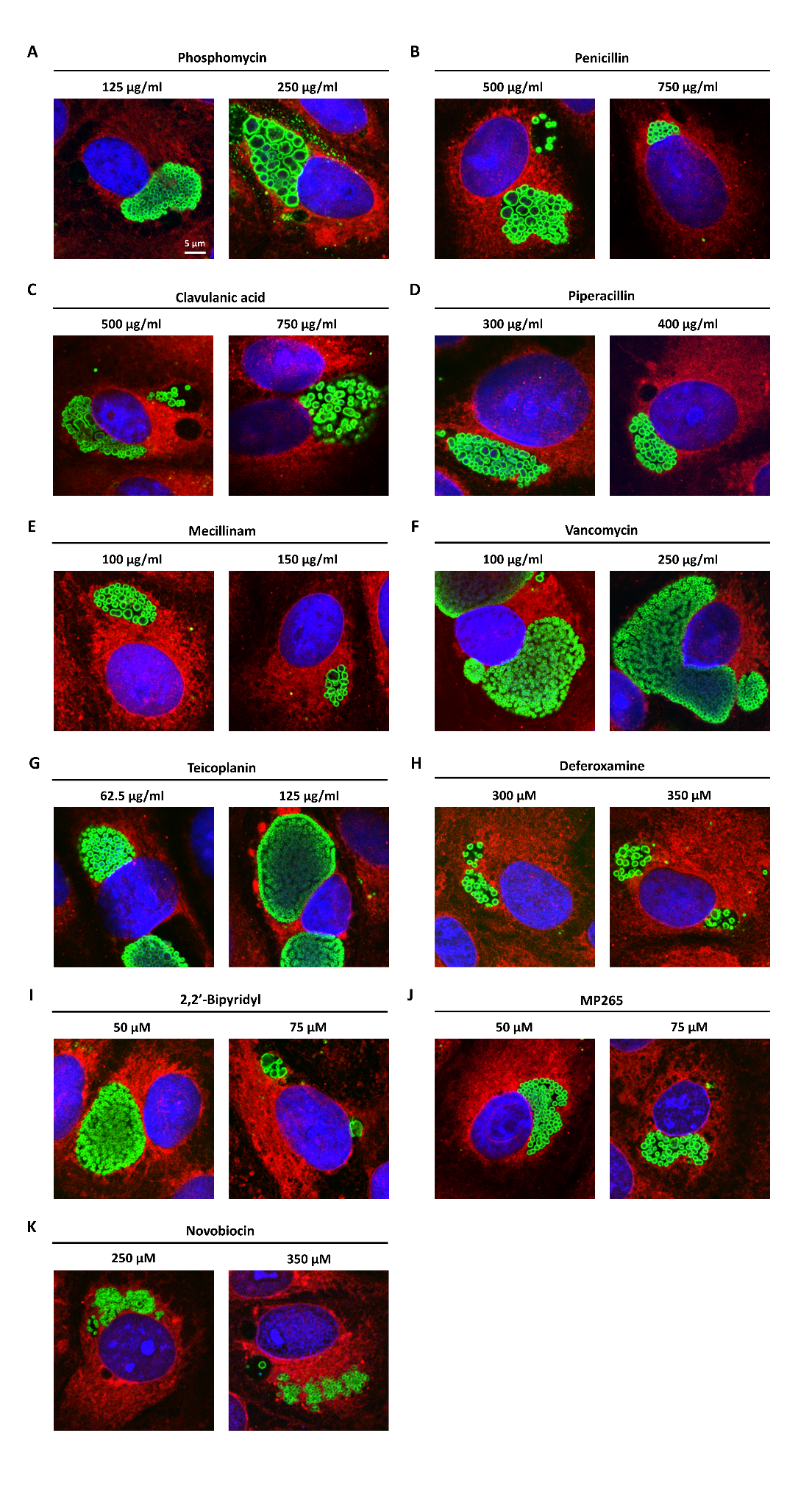
**

**Figure S3.** Determination of the optimal concentrations to induce formation of ABs. *W. chondrophila*-infected Vero cells were treated with the indicated drug concentrations at 2 hpi and fixed with methanol at 24 hpi (A-K). Infected Vero cells were stained with concanavalin A, DAPI and anti-*W. chondrophila* antibodies. All pictures display the same scale.

**
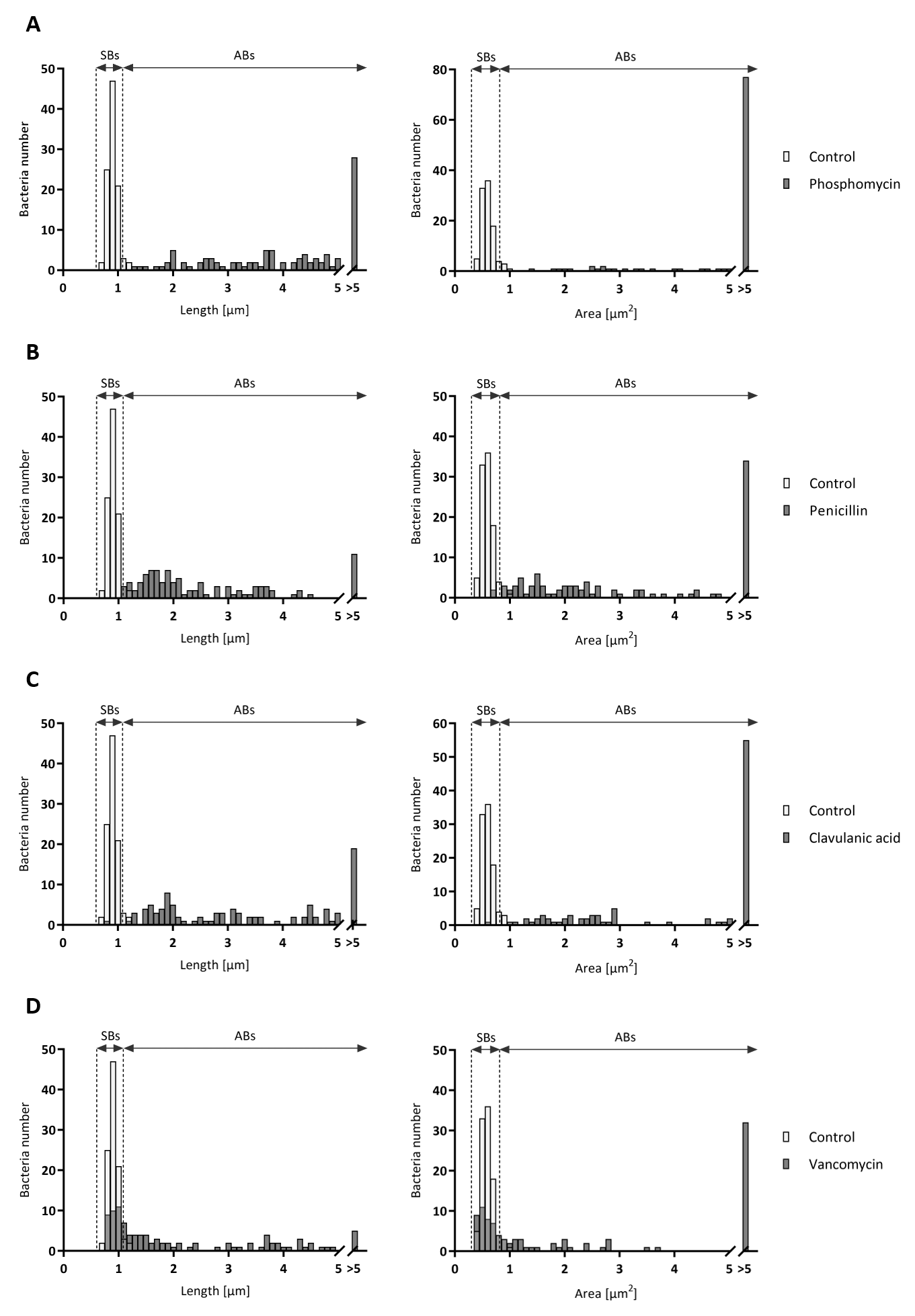
**

**
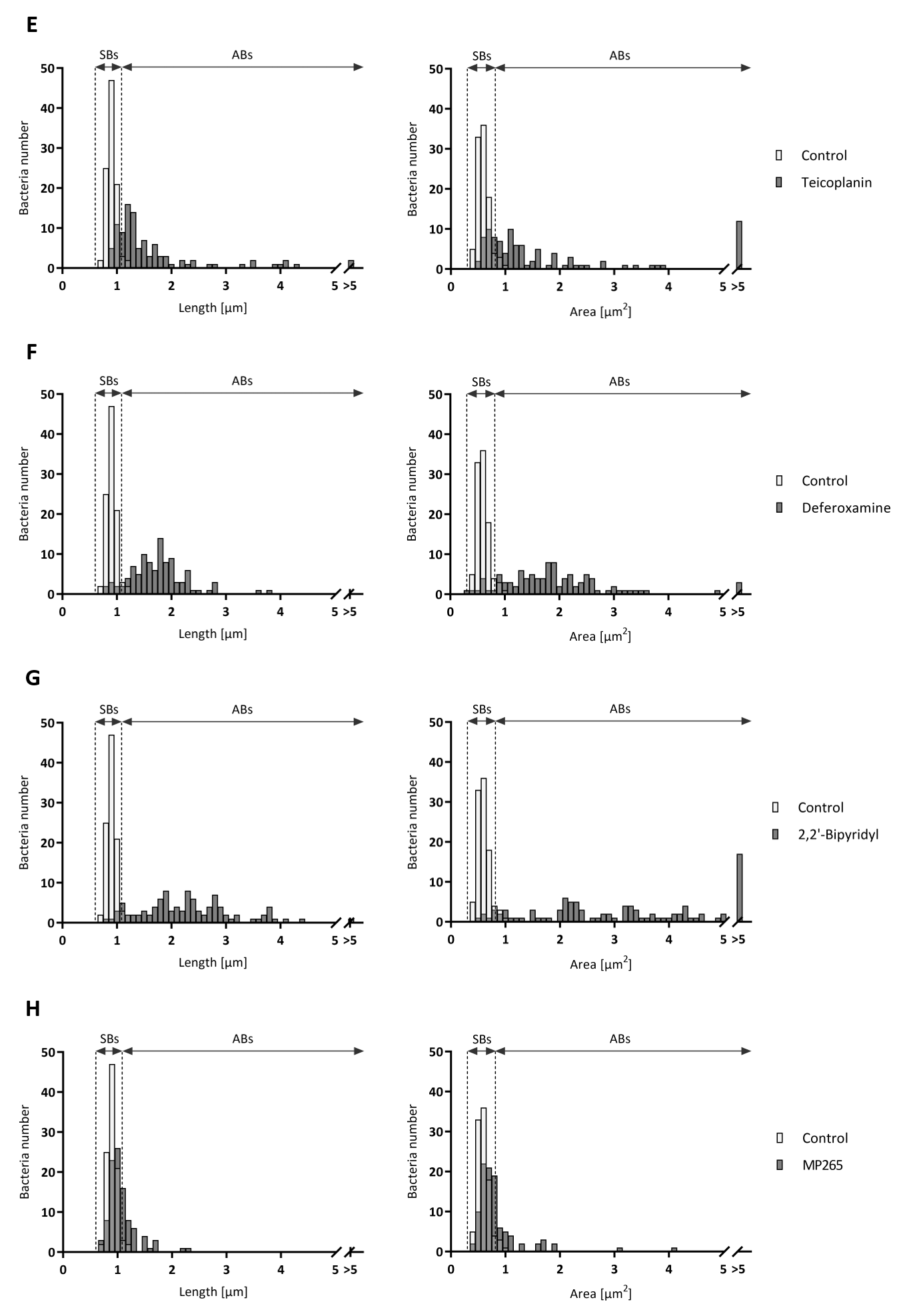
**

**
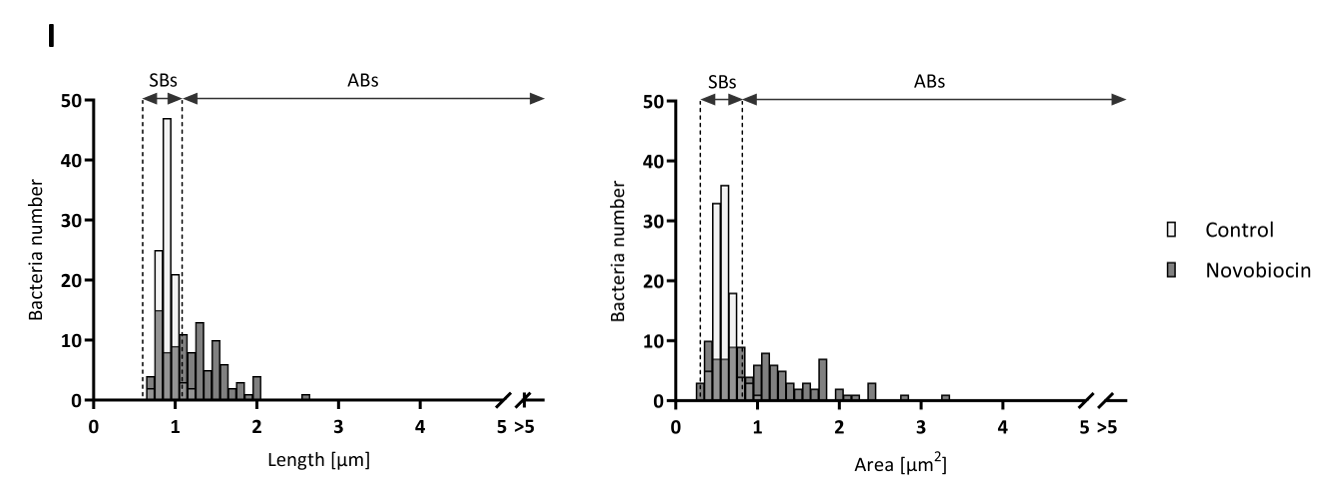
**

**Figure S4.** Frequency distribution of bacteria according to the length and area. The maximal length and area of 100 bacterial particles were measured from immunofluorescence pictures of Vero cells infected with *W. chondrophila*. Cells were treated with drug concentrations indicated in Table 1 at 2 hpi and fixed at 24 hpi. The cut-off (dotted line) between normal SBs and ABs represents the median + 2SD for length or area from the control sample. Images were analyzed with ImageJ. Frequency distribution between untreated and treated conditions were significantly different with a *P* value of <0.0001 for all drugs. Statistical analysis was performed with the Mann-Whitney test using Prism 7.03.

**Table S1. Primers and probes used in the study.**

| **Primers for qPCR** | |
| --- | --- |
| WadF4 [10] | 5’-GGCCCTTGGGTCGTAAAGTTCT-3’ |
| WadR4 [10] | 5’-CGGAGTTAGCCGGTGCTTCT-3’ |
| WadS2 [10] | 5’-FAM-CATGGGAACAAGAGAAGGATG-BHQ-3’ |
| **Primers for qRT-PCR** | |
| MreB_F [18] | 5'-CGCCTTGCCCGTCCCTAAGC-3' |
| MreB_R [18] | 5'-ATGGTTGTTGCCGGAGGCGG-3' |
| RodZ_F [18] | 5'-GCAGCTCATCTCTCCGGTTT-3' |
| RodZ_R [18] | 5'-CGCTCCAGGATTGCCTCTAG-3' |
