# Supplementary figures and images for: Diverse Stress-Inducing Treatments cause Distinct Aberrant Body Morphologies in the Chlamydia-Related Bacterium, Waddlia chondrophila
Source: Microorganisms. 2020 Jan 9;8(1):89. doi: 10.3390/microorganisms8010089 (PMC7022761; doi:10.3390/microorganisms8010089)

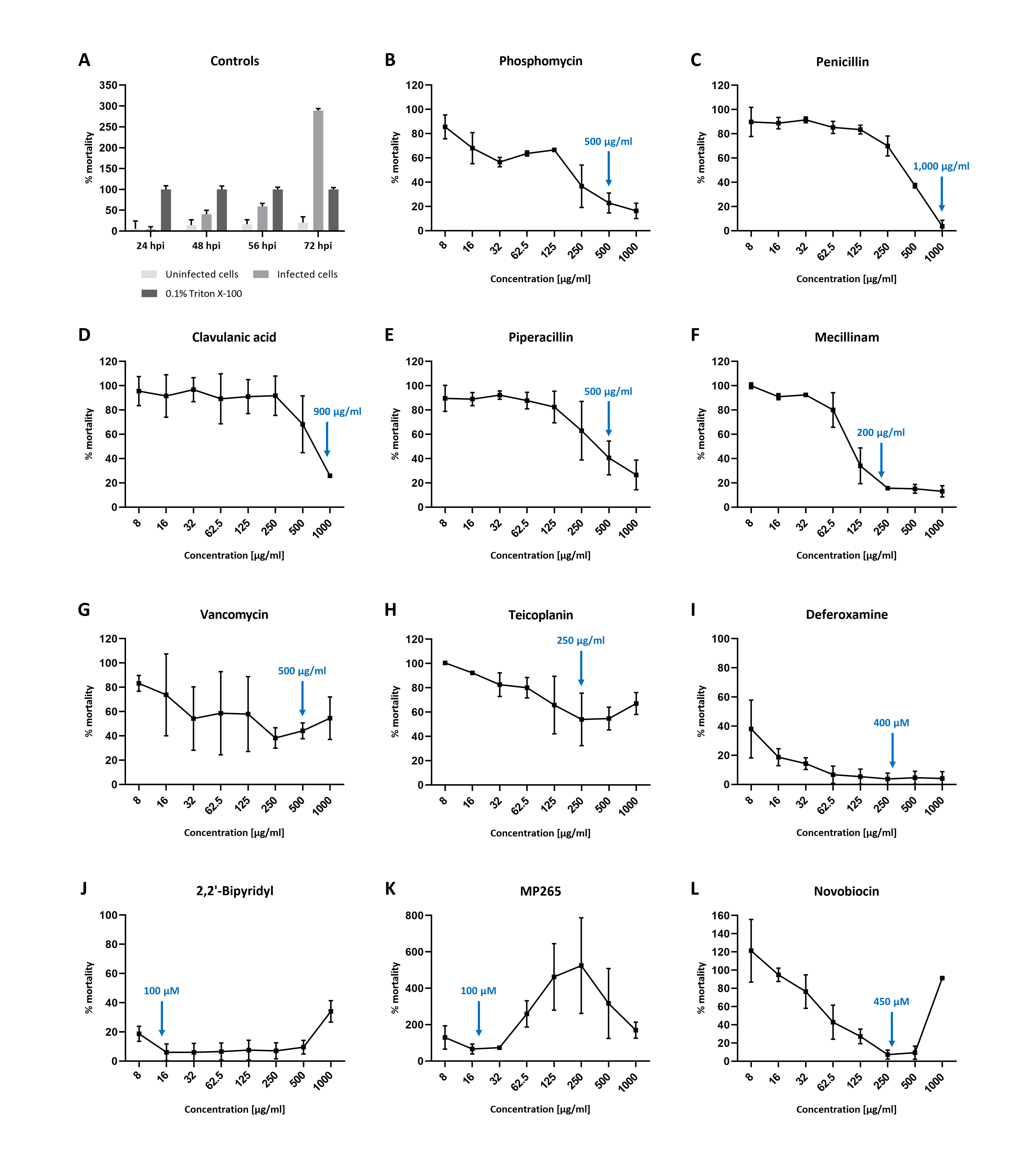

Supplement: Supplementary file 1 [file microorganisms-08-00089-s001.zip › Scherler_Supplementary_Data_Revised/Figure_S1.tif]

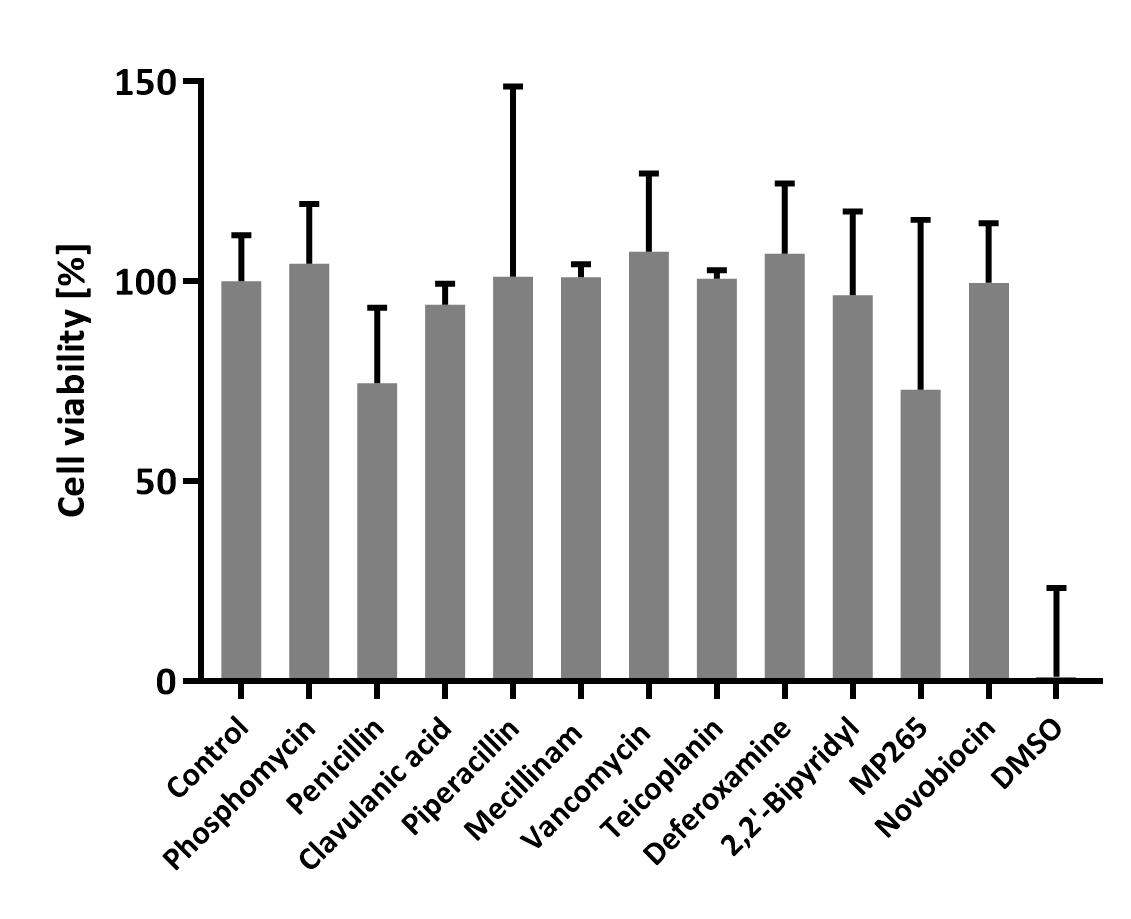

Supplement: Supplementary file 1 [file microorganisms-08-00089-s001.zip › Scherler_Supplementary_Data_Revised/Figure_S2.tif]

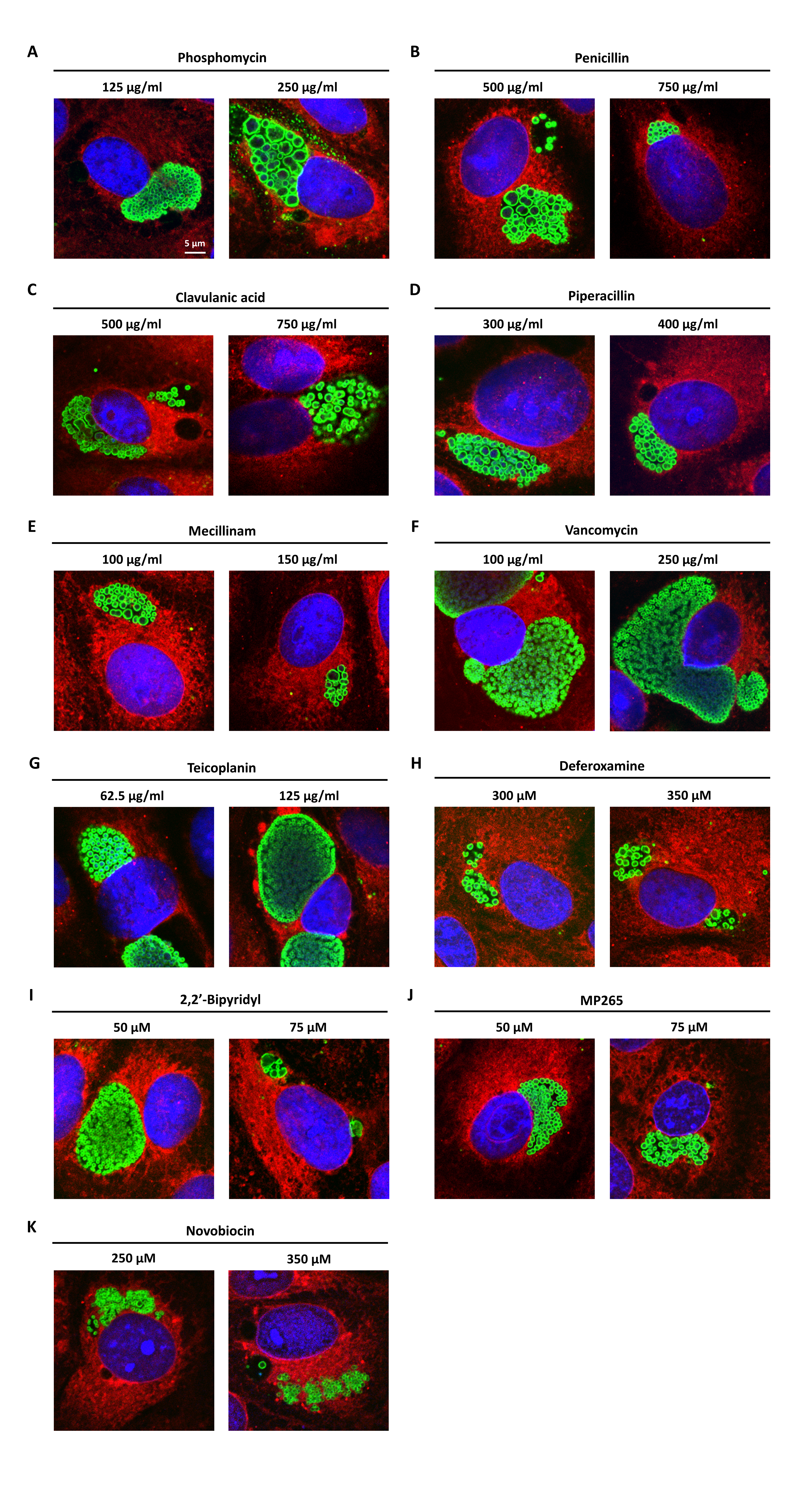

Supplement: Supplementary file 1 [file microorganisms-08-00089-s001.zip › Scherler_Supplementary_Data_Revised/Figure_S3.tif]

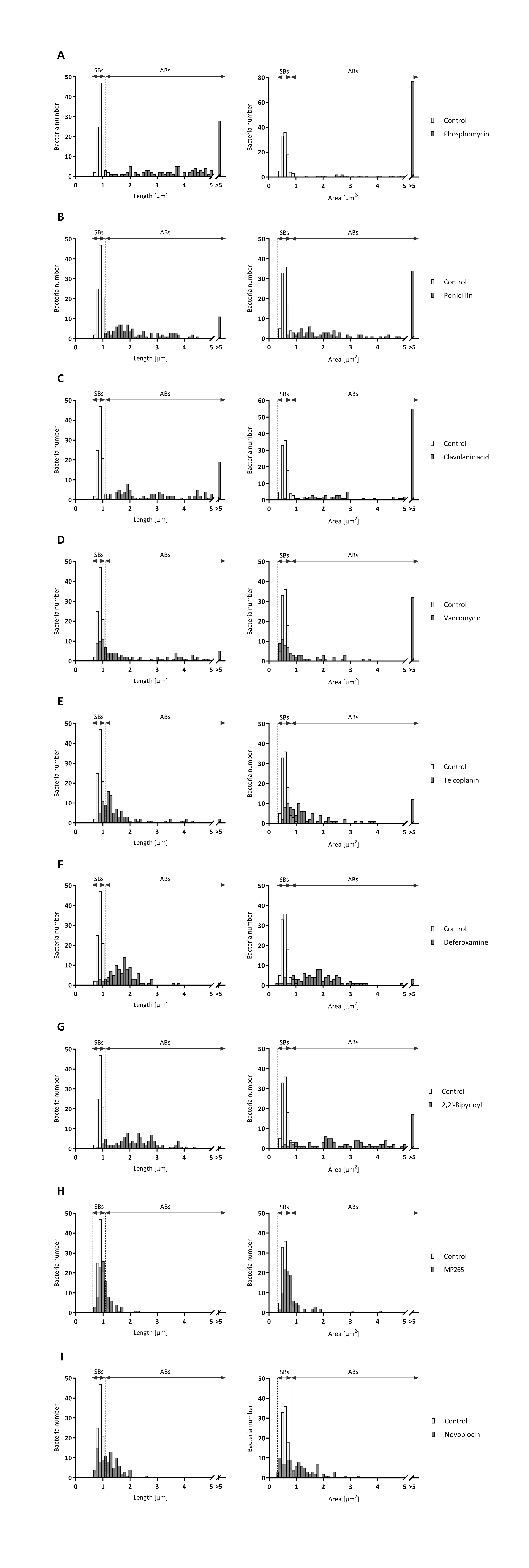

Supplement: Supplementary file 1 [file microorganisms-08-00089-s001.zip › Scherler_Supplementary_Data_Revised/Figure_S4_Revised.tif]
